# Dietary Omega-3 fatty acids inversely associated with systemic inflammatory response index (SIRI): a population-based analysis of NHANES 2005–2018

**DOI:** 10.3389/fnut.2025.1614427

**Published:** 2025-06-25

**Authors:** Xinglan Liang, Liangqin Luo, Juan Lu, Xiaoying Xie

**Affiliations:** ^1^Department of Nephrology, The Second Hospital of Longyan, Longyan, Fujian, China; ^2^Department of Neurosurgery, The Second Affiliated Hospital of Fujian Medical University, Quanzhou, Fujian, China; ^3^Department of Pediatrics, The Second Hospital of Longyan, Longyan, Fujian, China

**Keywords:** Omega-3 fatty acids, systemic inflammatory response index, NHANES, multifactorial logistic regression analysis, subgroup analysis

## Abstract

**Background:**

Omega-3 fatty acids are known for their anti-inflammatory and antioxidant properties. However, the relationship between Omega-3 intake and the systemic inflammatory response index (SIRI) remains unclear. This study aimed to examine the potential association between Omega-3 fatty acid intake and SIRI.

**Methods:**

A cross-sectional study was conducted using comprehensive data from the National Health and Nutrition Examination Survey (NHANES) for 2005–2018, assessing total Omega-3 fatty acid intake and SIRI among adults. SIRI was calculated using the formula monocyte × neutrophil count/lymphocyte count. The total dietary intake of Omega-3 fatty acids was calculated by summing the intakes of docosahexaenoic acid (DHA) and eicosapentaenoic acid (EPA). Subgroup analysis, smoothed curve fitting, and segmented linear regression were employed to investigate the relationship between SIRI and Omega-3 fatty acid consumption across genders.

**Results:**

A total of 26,416 participants were included in the study. Participants were classified into quartiles of Omega-3 fatty acid intake: 0–0.014, 0.015–0.037, 0.037–0.093, and 0.093–5.215. The participants’ SIRI ranged from 1.242 ± 0.916, with levels decreasing as Omega-3 fatty acid intake quartiles increased (Q1: 1.27 ± 0.88; Q2: 1.27 ± 1.01; Q3: 1.25 ± 0.91; Q4: 1.18 ± 0.87, *P* for trend < 0.001). In the fully adjusted model, total Omega-3 fatty acid consumption was negatively correlated with SIRI (β: −0.05; 95% CI: −0.09, −0.01). Subgroup analysis and interaction tests indicated no significant correlation between this negative association and age, sex, BMI, hypertension, diabetes mellitus, or coronary heart disease (*p* > 0.05 for all interactions). A “J”-shaped curve was observed in male participants, with an inflection point at 2.7 g Omega-3 fatty acid intake. On the left side of the inflection point, a negative correlation was observed (β: −0.07; 95% CI: −0.14, −0.00), whereas a positive and statistically significant correlation was found on the right side (β: 0.43; 95% CI: 0.05, 0.80; Logarithmic likelihood ratio test *P* = 0.014.

**Conclusion:**

A negative association may exist between SIRI and the consumption of omega-3 fatty. Further extensive studies are still needed to analyze their interaction.

## Introduction

The inflammatory response is a protective mechanism that the body employs in response to pathogen invasion and external environmental damage, involving processes such as the proliferation of inflammatory cells, the release of inflammatory mediators, and mobilization of immune responses. However, while protecting the organism, the inflammatory response can also lead to damage to the host organ, particularly under certain conditions (1–3). The primary goal of medical treatment is to enhance the host’s defense mechanisms and facilitate recovery, while also promoting the elimination of pathogens and mitigating external damage during the inflammatory response. This can be achieved through measures such as the appropriate use of antibiotics with minimal side effects and enhancing the body’s immune functions to eliminate foreign substances. In many cases, it is necessary to suppress the inflammatory response to prevent excessive damage to the organism (4).

Docosahexaenoic acid (DHA) and eicosapentaenoic acid (EPA), the two principal components of omega-3 polyunsaturated fatty acids, are naturally occurring anti-inflammatory agents predominantly found in fish and other human food sources. This study focuses on the relationship between omega-3 polyunsaturated fatty acids and the systemic inflammatory response. Most research on omega-3 fatty acids and inflammatory responses has focused on therapeutic studies related to various diseases. For instance, Al Biltagi et al. (5) found that omega-3 polyunsaturated fatty acids significantly improved lung function and controlled asthma, while Amer et al. (1), Tao et al. (3) reported that omega-3 fatty acid supplementation reduced all-cause mortality in cardiovascular diseases (6, 7). In the context of chronic kidney disease, Hu et al. (8) demonstrated the beneficial effects of omega-3 fatty acid supplementation. However, these studies do not explore the connection between omega-3 polyunsaturated fatty acids and the systemic inflammatory response at the organism level. The systemic inflammatory response index (SIRI), a novel index, reflects the overall level of systemic inflammation (9, 10). To date, no studies have reported the association between increased intake of omega-3 polyunsaturated fatty acids and decreased SIRI. However, the present study, using data from the NHANES database (2005–2018), demonstrates, for the first time, a strong association between these two variables.

## Subjects and methods

### Data and sample sources

This study utilizes data from the National Health and Nutrition Examination Surveys (NHANES), a research program administered by the National Center for Health Statistics (NCHS), a division of the Centers for Disease Control and Prevention (CDC). The objective of the program is to assess the nutritional and general health of the United States population through laboratory testing, physical examinations, and interviews. NHANES data is updated biennially. This study use stratified multistage random sample, ensuring representativeness. The publicly accessible website^1^ allows users to explore all the NHANES data used in this study.

**FIGURE 1:**
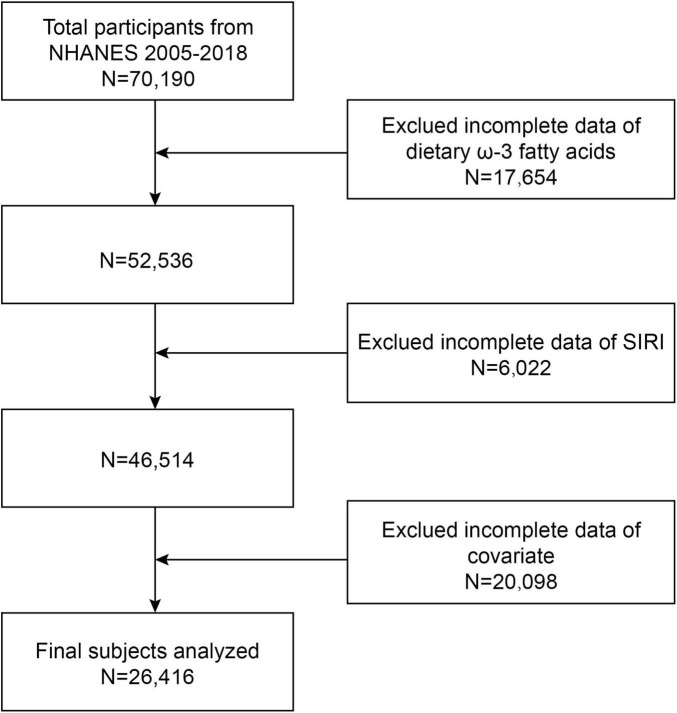
Flow chart. Exclued incomplete data of covariates included: hypertension (*n* = 13,209), diabetes (*n* = 14), coronary heart disease (*n* = 722), smoking (*n* = 3,036), education (*n* = 24), alcohol consumption (*n* = 20), marriage (*n* = 9), poverty ratio (*n* = 2,311), body mass index (BMI) (*n* = 259), high-density lipoprotein cholesterol (HDL-C) (*n* = 315), aspartate aminotransferase (AST) (*n* = 158), lanine aminotransferase (ALT) (*n* = 14), cholesterol (*n* = 4), and triglycerides (*n* = 3).

This study utilized data from the NHANES surveys conducted between 2005 and 2018, encompassing seven survey cycles. Initially, 70,190 participants were enrolled in the study and screened to exclude individuals with dietary deficiencies in omega-3 fatty acids (*n* = 17,654), absent SIRI data (*n* = 6,022), and missing data on other covariates (*n* = 20,098). Ultimately, data from 26,416 participants were analyzed (Figure 1).

### Exposure and outcome definitions

In this study, the total dietary intake of omega-3 fatty acids was calculated by summing the intake of EPA and DHA (11). Data on omega-3 component intake were collected using a food frequency questionnaire (FFQ) and a 24 h dietary recall method. The FFQ employed an automated multiple-pass method (AMPM) to ensure that the generated nutrient intake data did not deviate by more than 10% from actual intake (12). Participants recalled food and drink intake from the previous 24 h. Trained interviewers conducted the first dietary assessment, interviewing participants and collecting detailed dietary information for the preceding 24 h. A follow-up interview was scheduled by telephone 3–10 days later. The average of the two 24 h dietary recall surveys was used to calculate dietary omega-3 intake. The primary exposure variable in this study was omega-3 fatty acid intake.

Blood samples are given a complete blood cell count using the Beckman Coulter DxH 800 device in the NHANES Mobile Examination Center (MEC), which also distributes blood cells to each patient. According to a previous study, SIRI was calculated using the formula monocyte × neutrophil count/lymphocyte count (13). In this study, SIRI was used as an outcome variable.

### Covariates

In accordance with previous studies, the following covariates were considered in our analysis: age, gender, race, education level, household income-to-poverty ratio, marital status, body mass index (BMI), aspartate aminotransferase (AST), alanine aminotransferase (ALT), high-density lipoprotein cholesterol (HDL-C), blood cholesterol levels, blood triglyceride levels, alcohol consumption, smoking status, hypertension, diabetes, and coronary heart disease. Alcohol consumption was categorized as none, moderate (1–2 drinks/day), or excessive (three or more drinks/day) (14). Smoking status was defined as non-smoking or having smoked at least 100 cigarettes in a lifetime. Hypertension and coronary heart disease were categorized as “yes/no,” while diabetes mellitus was classified as “yes/no/preclinical diabetes” based on the questionnaire.

### Statistical analysis

The Student’s *t*-test was used for continuous data (reported as mean ± standard deviation), while the chi-square test was employed for categorical variables (reported as percentages). Multifactorial logistic regression analysis was utilized to investigate the relationship between SIRI and the dietary consumption of omega-3 fatty acids. Model 1 did not adjust for any covariates. Model 2 was partially adjusted for age, sex, and race. Model 3 was a fully adjusted model, accounting for age, sex, race, education level, marital status, household income-to-poverty ratio, body mass index (BMI), aspartate aminotransferase (AST), alanine aminotransferase (ALT), high-density lipoprotein cholesterol (HDL-C), cholesterol, triglycerides, alcohol consumption, smoking, hypertension, diabetes mellitus, and coronary heart disease. To further explore the relationship between omega-3 fatty acid intake and SIRI, smoothed curves were fitted, and two-stage linear regression was used to identify curve thresholds. Subgroup analyses based on gender, age, BMI, hypertension, coronary heart disease, and diabetes were also performed to assess the association between omega-3 fatty acid intake and SIRI in various subgroups. An interaction test was conducted to examine potential heterogeneity in the associations across subgroups. All analyses were performed using R software (version 4.1) and EmpowerStats (version 4.0).

## Results

In this study, a total of 26,416 participants were included, with 47.68% males and 52.32% females. The participants had a mean age of 49.54 ± 17.61 years. The omega-3 fatty acid intake was divided into four interquartile ranges: 0–0.014, 0.015–0.037, 0.037–0.093, and 0.093–5.215. The participants’ SIRI ranged from 1.242 ± 0.916. Moreover, as dietary omega-3 fatty acid intake quartiles increased, SIRI levels progressively decreased (Q1: 1.27 ± 0.88; Q2: 1.27 ± 1.01; Q3: 1.25 ± 0.91; Q4: 1.18 ± 0.87, *p* < 0.001). Based on the Omega-3 fatty acid intake quartiles, the main characteristics of the population with higher Omega-3 fatty acid intake were older married men, moderate alcohol consumption, non-smokers, income and education level, higher HDL cholesterol, aspartate aminotransferase levels, and low triglyceride levels (Table 1).

**TABLE 1:** Baseline characteristics of the study population.

ω-3 fatty acids (g)	Q1 (0–0.014)	Q2 (0.015–0.037)	Q3 (0.037–0.093)	Q4 (0.093–5.215)	*P*-value
	***N* = 6,518**	***N* = 6,629**	***N* = 6,658**	***N* = 6,611**	
Age, years	49.88 ± 18.18	49.14 ± 18.02	49.05 ± 17.34	50.09 ± 16.87	< 0.001
Poverty income ratio	2.43 ± 1.60	2.49 ± 1.60	2.58 ± 1.61	2.79 ± 1.66	< 0.001
BMI (kg/m^2^)	29.08 ± 6.86	29.48 ± 7.05	29.67 ± 7.05	29.20 ± 6.93	< 0.001
HDL-C (mg/dL)	52.99 ± 16.14	53.01 ± 15.95	52.77 ± 15.69	53.96 ± 16.32	< 0.001
ALT (U/L)	24.15 ± 23.63	24.49 ± 18.34	25.68 ± 20.99	25.65 ± 20.27	< 0.001
AST (U/L)	25.22 ± 18.54	25.13 ± 17.69	25.47 ± 18.10	25.78 ± 16.46	< 0.001
Cholesterol (mg/dL)	193.86 ± 42.84	194.26 ± 42.06	193.26 ± 41.24	194.23 ± 41.69	0.47
Triglycerides (mg/dL)	154.23 ± 115.87	154.21 ± 119.40	152.61 ± 114.83	151.98 ± 132.06	< 0.001
SIRI	1.27 ± 0.88	1.27 ± 1.01	1.25 ± 0.91	1.18 ± 0.87	< 0.001
Gender, %	–	–	–	–	< 0.001
Male	2,692 (41.30%)	2,960 (44.65%)	3,442 (51.70%)	3,502 (52.97%)	–
Female	3,826 (58.70%)	3,669 (55.35%)	3,216 (48.30%)	3,109 (47.03%)	–
Race, %	–	–	–	–	< 0.001
Mexican American	852 (13.07%)	975 (14.71%)	1,169 (17.56%)	966 (14.61%)	–
Other Hispanic	580 (8.90%)	595 (8.98%)	613 (9.21%)	566 (8.56%)	–
Non-Hispanic White	3,390 (52.01%)	3,301 (49.80%)	2,841 (42.67%)	2,542 (38.45%)	–
Non-Hispanic Black	1,064 (16.32%)	1,227 (18.51%)	1,489 (22.36%)	1,636 (24.75%)	–
Other races	632 (9.70%)	531 (8.01%)	546 (8.20%)	901 (13.63%)	–
Education level, %	–	–	–	–	< 0.001
Less than junior high school	607 (9.31%)	599 (9.04%)	591 (8.88%)	538 (8.14%)	–
Middle to high school	961 (14.74%)	866 (13.06%)	918 (13.79%)	809 (12.24%)	–
High school graduate/GED or equivalent	1,554 (23.84%)	1,577 (23.79%)	1,557 (23.39%)	1,357 (20.53%)	–
Some college or AA degree	1,972 (30.25%)	2,081 (31.39%)	2,009 (30.17%)	1,954 (29.56%)	–
College graduate or above	1,424 (21.85%)	1,506 (22.72%)	1,583 (23.78%)	1,953 (29.54%)	–
Marital status, %	–	–	–	–	< 0.001
Married	3,336 (51.18%)	3,443 (51.94%)	3,618 (54.34%)	3,651 (55.23%)	–
Widowed	574 (8.81%)	523 (7.89%)	457 (6.86%)	428 (6.47%)	–
Divorced	775 (11.89%)	717 (10.82%)	673 (10.11%)	742 (11.22%)	–
Separated	212 (3.25%)	220 (3.32%)	209 (3.14%)	218 (3.30%)	–
Never married	1,107 (16.98%)	1,183 (17.85%)	1,142 (17.15%)	1,089 (16.47%)	–
Living with partner	514 (7.89%)	543 (8.19%)	559 (8.40%)	483 (7.31%)	–
Alcohol consumption, %	–	–	–	–	< 0.001
No	2,534 (38.88%)	2,330 (35.15%)	2,299 (34.53%)	2,129 (32.20%)	–
1–2 cups per day	2,607 (40.00%)	2,742 (41.36%)	2,713 (40.75%)	2,988 (45.20%)	–
More than 2 cups per day	1,377 (21.13%)	1,557 (23.49%)	1,646 (24.72%)	1,494 (22.60%)	–
Diabetes, %	–	–	–	–	0.003
No	5,588 (85.73%)	5,678 (85.65%)	5,606 (84.20%)	5,596 (84.65%)	–
Yes	812 (12.46%)	778 (11.74%)	897 (13.47%)	854 (12.92%)	–
Borderline	118 (1.81%)	173 (2.61%)	155 (2.33%)	161 (2.44%)	–
Coronary heart disease, %	–	–	–	–	0.218
No	6,218 (95.40%)	6,367 (96.05%)	6,373 (95.72%)	6,347 (96.01%)	–
Yes	300 (4.60%)	262 (3.95%)	285 (4.28%)	264 (3.99%)	–
Smoking status, %	–	–	–	–	0.002
No	3,509 (53.84%)	3,654 (55.12%)	3,648 (54.79%)	3,777 (57.13%)	–
At least 100 cigarettes in life	3,009 (46.16%)	2,975 (44.88%)	3,010 (45.21%)	2,834 (42.87%)	–
Hypertension, %	–	–	–	–	0.703
No	4,185 (64.21%)	4,272 (64.44%)	4,277 (64.24%)	4,199 (63.52%)	–
Yes	2,333 (35.79%)	2,357 (35.56%)	2,381 (35.76%)	2,412 (36.48%)	–

HDL-C, high-density lipoprotein-cholesterol; ALT, alanine aminotransferase; AST, aspartate aminotransferase; BMI, body mass index; SIRI, systemic inflammatory response index.

### Connection of Omega-3 fatty acid intake with SIRI

In the unadjusted model (Model 1), we found a negative association between overall omega-3 intake and SIRI (β: −0.10; 95% CI: −0.14, −0.05) (Table 2). Dietary Omega-3 intake remained negatively associated with SIRI when partially adjusted for potential confounders (Model 2: β: −0.08; 95% CI: −0.12, −0.04), and the association persisted in the fully adjusted model (Model 3: β: −0.05; 95% CI: −0.09, −0.01). In the fully adjusted model, SIRI levels decreased by 0.05 for each 1 g increase in omega-3 intake. To further validate the findings, dietary omega-3 consumption was converted from a continuous to a categorical variable. In the fully adjusted model, SIRI levels were significantly reduced by 0.041 (β: −0.041; 95% CI: −0.071, −0.010) in participants in the highest quartile (Q4) compared with those in the lowest quartile (Q1) of omega-3 intake, and this reduction was statistically significant (*P* for trend < 0.00058).

**TABLE 2:** Correlation between Omega-3 fatty acid intake and systemic inflammatory response index (SIRI).

Omega-3 fatty acid intake	β (95% CI)		
	**Crude model (Model 1)**	**Minimally adjusted model (Model 2)**	**Fully adjusted model (Model 3)**
Continuous	−0.10 (−0.14, −0.05) < 0.0001	−0.08 (−0.12, −0.04) 0.0004	−0.05 (−0.09, −0.01) 0.0277
Categories	–	–	–
Q1	Reference	Reference	Reference
Q2	−0.000 (−0.031, 0.031) 0.99338	0.005 (−0.025, 0.036) 0.74082	0.008 (−0.022, 0.038) 0.60055
Q3	−0.014 (−0.045, 0.017) 0.37931	0.001 (−0.029, 0.032) 0.93312	0.006 (−0.025, 0.036) 0.70890
Q4	−0.085 (−0.116, −0.054) < 0.00001	−0.059 (−0.090, −0.028) 0.00017	−0.041 (−0.071, −0.010) 0.00897
*P* for trend	< 0.00001	< 0.00001	0.00058

Model 1, no covariates were adjusted; Model 2, adjusted for sex, age, and race; Model 3, adjusted for all covariates. β, regression coefficient.

### Non-linear relationship between Omega-3 fatty acid intake and SIRI

**FIGURE 2:**
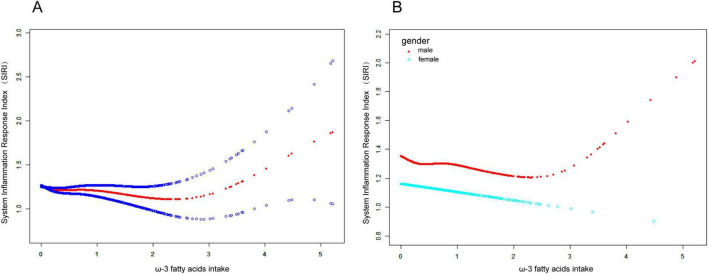
Connection between Omega-3 fatty acid intake and SIRI. **(A)** The smoothing of the fit is indicated by the red curve, and the 95% confidence interval is indicated by the blue curve. There is a non-linear correlation between Omega-3 fatty acid intake and SIRI. **(B)** Association between Omega-3 fatty acid intake and SIRI stratified by gender. Omega-3 fatty acid intake showed a “J”-shaped curve relationship with SIRI in male participants, but a linear positive relationship in female patients.

To explore the potential non-linear relationship between dietary omega-3 fatty acid intake and SIRI, smoothed curve fitting was performed. The results in Figure 2A show a significant non-linear relationship between dietary omega-3 fatty acid intake and SIRI. Further smoothed curve fitting, stratified by gender (Figure 2B), revealed a “J”-shaped curve between dietary omega-3 fatty acid intake and SIRI in male participants, while a linear relationship was observed in female participants. Segmented linear regression was then applied to further validate this non-linear relationship. In the male population, the inflection point of the “J”-shaped curve occurred at 2.7 g. On the left side of the inflection point, a negative correlation between SIRI and omega-3 fatty acid intake was observed (β: −0.07; 95% CI: −0.14, −0.00), whereas a positive correlation was found on the right side (β: 0.43; 95% CI: 0.05, 0.80) (Logarithmic likelihood ratio test *P* = 0.014). In contrast, no non-linear relationship was observed in female participants (Logarithmic likelihood ratio test *P* = 0.365) (Table 3).

**TABLE 3:** Threshold effect analysis of Omega-3 fatty acid intake and systemic inflammatory response index (SIRI).

		Male	Female	Total
Model	β (95% CI)	−0.04 (−0.10, 0.03)	−0.06 (−0.12, 0.00)	−0.05 (−0.09, −0.01)
	*P* for trend	0.2631	0.0585	0.0277
Model 3	Breakpoint (K)	2.7	2.7	2.7
	β1 (< K)	−0.07 (−0.14, −0.00)	−0.07 (−0.13, −0.00)	−0.07 (−0.12, −0.03)
	*P* for trend	0.0359	0.0402	0.0023
	β2 (> K)	0.43 (0.05, 0.80)	0.31 (−0.49, 1.10)	0.38 (0.06, 0.69)
	*P* for trend	0.0255	0.4518	0.0196
	Logarithmic likelihood ratio test *P*	0.014	0.365	0.008

Model, contrast model; Model 3, adjusted for all the covariates except gender.

### Subgroup analysis

**FIGURE 3:**
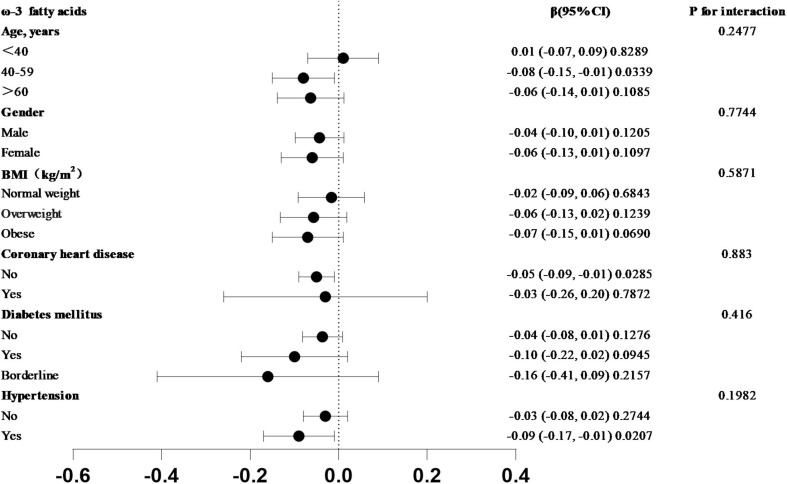
Subgroup analysis of the association between Omega-3 fatty acid intake and SIRI in forest plot. In the subgroups of age, sex, BMI, coronary heart disease, hypertension, and diabetes mellitus, which trend test *p*-value greater than 0.05.

The correlation between omega-3 fatty acid intake and SIRI levels varied across the subgroups analyzed (Figure 3). In the subgroups based on age, sex, BMI, coronary heart disease, hypertension, and diabetes mellitus, a stronger correlation between dietary omega-3 fatty acid intake and SIRI was observed in participants aged 40–59 years, females, those with obesity, individuals without coronary heart disease, those with preclinical diabetes, and participants with hypertension. To assess whether this relationship held across the entire population, an interaction test was conducted. The results indicated that the negative association between omega-3 fatty acid intake and SIRI levels was independent of subgroups based on age, sex, BMI, coronary heart disease, hypertension, and diabetes mellitus (all *P* for interaction > 0.05).

## Discussion

This study examined data from seven cycles of the NHANES survey (2005–2018) in a cross-sectional analysis. To date, this study is the first to report an association between increased dietary intake of omega-3 polyunsaturated fatty acids and lower SIRI levels. To control for confounding factors, age, gender, and race were included as covariates in Model 2, while Model 3 was fully adjusted, incorporating all relevant factors, and yielded similar results. Omega-3 polyunsaturated fatty acid intake was divided into quartiles, with Q1 as the reference group. A test for trend was performed, and the *P*-values for trend in all three models were less than 0.05. A strong negative correlation was observed between omega-3 polyunsaturated fatty acid intake and SIRI.

Systemic inflammatory response index is a systemic inflammatory response index that reflects the overall severity of inflammation in the body (15). Inflammation serves as a fundamental defense mechanism against infection and injury, involving the activation of immune cells and release of cytokines such as TNF-α and IL-6 (16). While normally self-limiting, inflammation can become chronic due to metabolic dysregulation, contributing to tissue damage in conditions such as atherosclerosis and obesity (17, 18). In these cases, patients often show immune cell infiltration in tissues and elevated systemic inflammatory mediators, though anti-inflammatory medications are not routinely used. Instead, dietary strategies, particularly those involving anti-inflammatory nutrients, are gaining attention. Omega-3 polyunsaturated fatty acids (PUFAs), notably DHA and EPA, have been widely studied for their role in modulating inflammatory pathways. Recent research suggests that higher omega-3 intake may help reduce systemic inflammation by limiting leukocyte activation, decreasing pro-inflammatory cytokine release, and stabilizing cell membranes (19). In diseases such as cardiovascular disorders, rheumatoid arthritis, and asthma, omega-3 PUFAs have shown beneficial adjunctive effects. Given these findings, dietary omega-3 intake may represent a feasible approach to mitigating chronic inflammation in the general population.

The total dietary intake of omega-3 fatty acids was calculated by summing the intake of DHA and EPA (20). Numerous recent studies have confirmed the anti-inflammatory effects of omega-3 polyunsaturated fatty acids. A study by Zhang et al. (21) supplemented the diet of patients with rheumatoid arthritis with omega-3 polyunsaturated fatty acids, significantly relieving symptoms. Zeyda et al. (22) demonstrated that omega-3 polyunsaturated fatty acids can inhibit inflammatory responses and exert anti-inflammatory effects in rats by regulating prostatic transmembrane protein-2 (STAMP2) in animal models. In a cross-sectional study of chronic kidney disease, the addition of omega-3 polyunsaturated fatty acid-rich fish oil to the diet was shown to complement medical therapy, alleviating symptoms and prolonging survival by reducing the degree of inflammatory response (23). Several recent studies on the anti-inflammatory mechanisms of omega-3 polyunsaturated fatty acids suggest the following possible mechanisms: First, omega-3 polyunsaturated fatty acids inhibit the proliferation of leukocytes and reduce their accumulation at the site of inflammation by altering the secretion of leukocyte adhesion factors in the vascular wall, thereby decreasing the local inflammatory response. Second, the chemotaxis of leukocytes reduces their migration from the lesion site. Leukocytes respond to the release of chemicals at the site of inflammation by migrating toward them through a mechanism called chemotaxis. These substances, including the eicosanoids LTB4 produced from arachidonic acid, are known as chemoattractants. Studies in healthy human subjects supplemented with fish oil rich in omega-3 polyunsaturated fatty acids have shown that reduced leukocyte chemotaxis to various chemoattractants leads to a decrease in the production of inflammatory factors such as IL-6, IL-1β, and TNF-α. Third, the anti-inflammatory effect of omega-3 polyunsaturated fatty acids is also attributed to their protection of cell membranes from damage at the site of inflammation. Omega-3 fatty acids can reduce the secretion of arachidonic acid through competitive binding, stabilizing the phospholipid structure of the cell membrane and protecting cells from inflammation (24). In conclusion, omega-3 polyunsaturated fatty acids are associated with lower SIRI levels, suggesting potential anti-inflammatory properties, and the incorporation of omega-3 fatty acid-rich foods into the diet can improve the level of inflammation in the organism’s internal environment, thereby protecting the organism.

Subgroup analyses were conducted across different populations to assess the stability of the model associating omega-3 polyunsaturated fatty acids with SIRI. The results revealed that the negative association between omega-3 polyunsaturated fatty acid intake and SIRI levels was independent of age, sex, BMI, coronary heart disease, hypertension, and diabetes mellitus subgroups (*P* for interaction > 0.05). No significant differences were observed in the intake of adequate fish-based diets across age and gender. In diabetic patients, increasing the intake of fish rich in omega-3 polyunsaturated fatty acids reduces complications and improves prognosis, particularly the incidence of diabetic nephropathy (23). Several recent studies on cardiovascular disease treatment have demonstrated that omega-3 polyunsaturated fatty acids improve prognosis and quality of life (25–27). According to dietary guidelines for individuals with obesity, foods high in omega-3 polyunsaturated fatty acids from fish should be consumed regularly over an extended period. These findings align with the subgroup analysis of the present study, where the negative correlation between omega-3 polyunsaturated fatty acid intake and SIRI levels remained stable across different populations.

Our study has several strengths. First, the large, nationally representative sample enhanced the statistical robustness and reliability of our results. Second, this study is the first to evaluate the dose-response relationship between dietary omega-3 fatty acid intake and SIRI. Third, we adjusted for several potential confounders in our exploration of the association between omega-3 fatty acid intake and SIRI. However, our study has several limitations. First, establishing causality is challenging due to the cross-sectional design of this study. Additionally, despite adjusting for several variables, confounding bias from unmeasured factors cannot be entirely ruled out. Third, nutritional data were collected through two 24 h dietary recall interviews; however, these interviews may not fully capture an individual’s average intake. Finally, the conclusions of this study apply to the United States adult population for which data on omega-3 fatty acid intake are available, and the results should not be generalized to individuals who lack relevant dietary data or to populations in other countries and regions.

## Conclusion

In conclusion, the results of this study indicate a negative correlation between omega-3 polyunsaturated fatty acid intake and SIRI levels. This negative association remains significant even after adjusting for potential confounders, including age, sex, and BMI. Our findings support the hypothesis that omega-3 fatty acids are anti-inflammatory effects and may contribute to reducing systemic inflammation. Further longitudinal studies are needed to clarify the mechanisms linking omega-3 intake and systemic inflammation. Additionally, further research is necessary to explore the optimal dosage and long-term effects of omega-3 fatty acid supplementation in diverse populations.

## Data Availability

The raw data supporting the conclusions of this article will be made available by the authors, without undue reservation.

## Footnotes

1
www.cdc.gov/nchs/nhanes/
